# Issue framing in online voting advice applications: The effect of left-wing and right-wing headers on reported attitudes

**DOI:** 10.1371/journal.pone.0212555

**Published:** 2019-02-21

**Authors:** Naomi Kamoen, Jasper van de Pol, André Krouwel, Claes de Vreese, Bregje Holleman

**Affiliations:** 1 Department of Communication and Information Sciences, Tilburg University, Tilburg, the Netherlands; 2 Amsterdam School of Communication Research (ASCoR), University of Amsterdam, Amsterdam, the Netherlands; 3 Department of Communication, VU University, Amsterdam, the Netherlands; 4 Utrecht Institute of Linguistics, Utrecht University, Utrecht, the Netherlands; Sogang University (South Korea), REPUBLIC OF KOREA

## Abstract

Voting Advice Applications (VAAs) provide voting recommendations to millions of people. As these voting recommendations are based on users’ answers to attitude questions, the framing of these questions can have far-reaching consequences. The current study reports on a field experiment in which the framing of the header above VAA statements (*N* = 17) was manipulated (condition 1: no header; condition 2: a right-wing header, e.g., *finance*; condition 3: a left-wing header, e.g., *nature and environment*). Visitors of a VAA developed for Utrecht, the fourth largest municipality in the Netherlands, were randomly guided to one of the versions of the tool in which the header type was varied. Results (based on *N*_*respondents*_ = 27,404) show that providing a header (left-wing or right-wing) leads to more left-wing answers as compared a condition where there is no header above the attitude statement. This effect, however, is only observed for respondents with lower levels of political sophistication.

## Introduction

Framing can shape public opinion through individuals’ perceptions of political issues and choices in many ways. Voting Advice Applications (VAAs) form an important context in which framing effects can have direct and far-reaching consequences. Before casting their ballot, a large and increasing number of voters visit these web applications in order to receive voting advice [1]. VAAs ask their users to report their attitudes on a set of policy statements. An algorithm then compares users’ attitudes to party positions, after which the VAA tells its users which party matches their issue positions best, and to what extent users agree with all other competing parties. Most of these VAAs claim to provide a neutral and balanced overview of the issue agreement with each party [2], [3]. Since the VAA recommendations are based on reactions to policy statements, the framing of these statements potentially impacts the attitudes people report, and hence the voting advice received by a substantial share of the electorate.

In various other political attitude contexts, it has already been shown that the framing of attitude questions can greatly affect people’s attitudes [4]. More people, for instance, are willing to tolerate a Ku Klux Klan meeting if this is framed as a “free speech issue” than when framed as “a threat to public order” [5]; more egalitarians agree with government support for poor people if that action is framed as giving them a “better chance of getting ahead” rather than when it is framed as “leading to higher taxes” [6]; and “climate change” is considered an actual phenomenon by more Republicans than “global warming” is [7].

The attitude questions or statements included in Voting Advice Applications have the same potential of steering the attitudes people report to them. Each policy issue is captured in a statement of one or two sentences, often accompanied by a header classifying the policy theme of the issue in a few keywords. If a particular frame directs people’s attitudes in a certain direction, this will likely affect the voting advice that people receive as this advice is based directly on the answers provided [8]. Since several studies found evidence of a persuasive effect of this voting advice on people’s actual vote choices (e.g., [9], [10], [11]), studying the potential effect of framing in this particular context of language in use is of utmost importance. The fact that VAAs are frequented by large numbers of people–up to 52% of voters during Dutch national elections (*Stemwijzer*), 30% in Germany (*Wahl-O-Mat*), 36% in Finland (*Vaalikone*), and 30% in Sweden (*Valkompassen*) [1]—stresses the urgency of studying framing effects in VAAs once more. Moreover, this specific real-life context allows for the use of subtle framing manipulations that only measure the emphasis placed on a certain aspect of the question rather than also manipulating the content or number of arguments, as has frequently been done in earlier framing studies [12]. Therefore, we aim to answer the following research question: *To what extent does the way headers are framed in VAAs affect the attitudes people report towards these issues*? As it is often suggested that the size of framing effects will depend on the extent to which language users hold existing attitudes to work from ([13], [14], [15]), we also test the potentially moderating effect of political sophistication on people’s reported attitudes in VAAs.

### Issue framing

VAAs are intended to make politics more comprehensible for the general public (e.g., [2], [16]). The way political issues are perceived depends on the problem definitions that are put forward by different frames [17]. When VAA developers formulate issue statements, inevitably they offer users a frame. For instance, consider the positively phrased statement, “The nuclear power station in Borssele should stay open” [18]. This formulation is likely to activate different associations and considerations compared to an alternative negative statement, such as “The nuclear power station in Borssele should be closed” (see [19] for a discussion of polarity effects). Likewise, the header above the VAA statements can also carry a certain problem definition. The statement about the nuclear power station can for example be placed under the heading *Environment*, as well as under *Energy supply*. The choice for either one of these frames probably highlights different aspects of the issue.

Different headers and different ways of phrasing attitude statements are examples of framing. Over the past decades, scholars have gathered a vast amount of evidence of such framing choices affecting public opinion (e.g, [4], [20], [21]). Within political communication, these framing effects have been classified into two broad categories (see [21], [22], [23], [24]). *Equivalence framing*, often found in psychological research and behavioral economics literature, exists of “two logically equivalent (but not transparently equivalent) statements of a problem [that] lead decision makers to different options” ([25]: 36). The famous experiment by Tversky and Kahneman [26] is often cited in this context, which demonstrated that people are inclined to be risk averse if a dilemma is framed in terms of gains and tend to be risk taking if the same dilemma is framed in terms of losses. Positive and negative wording alternatives for policy statements, like the one above about the nuclear power station at Borssele, are another example of equivalence framing.

The second category of framing research is called *emphasis framing*, and is often studied within the field of media effects. Here, a more relaxed definition of framing is applied, supposedly better reflecting the “complexity of everyday communication environments, where attitude formation is likely driven by an interplay of complementary or competing frames” ([24]: 5). Many studies on forms of emphasis framing use the definition of framing by Gamson and Modigliani ([27]: 143), who define framing to be a “central organizing idea or story line that provides meaning to an unfolding strip of events, weaving a connection among them. The frame suggests what the controversy is about, the essence of the issue.” For example, [28] found that a news story framing the entry of Bulgaria and Romania to the EU as a financial opportunity resulted in a more positive opinion towards the economic consequences of this choice as compared to the same news story emphasizing the monetary risks.

Literature on emphasis framing has often been criticized for using a definition of framing which is *too* relaxed and inclusive (see e.g., [17], [29], [30]). First of all, emphasis framing operationalizations are context-specific which decreases the generalizability of potential framing effects. Second, and more importantly, emphasis framing studies have been criticized because they confound real emphasis framing, that is, placing emphasis on a specific topic, with different or additional information about that topic [12]. [31] demonstrated in a meta-analysis that when controlling for the amount and content of information the effects of emphasis framing are very limited.

In order to meet these two points of criticism, the current field experiment investigates the effect of changing the header that comes with the VAA question, consistently comparing a condition with no header above the question to a second condition with a right-wing frame (e.g., finance) and a third condition with a left-wing frame (e.g., nature and environment). This way we study a very minimalist form of emphasis framing that is not prone to the critique that more information is offered.

Second, we aim to generalize our findings beyond the level of the individual item by applying the same type of left-wing and right-wing frames across items. This left-right continuum is the dominant way political views are organized in many Western societies (e.g., [32], [33]), and it is (therefore) also used in VAA practice to classify the statements and to organize the voting advice [8]. The left-right distinction is also apparent in a citizen’s organization of political information; e.g., [34] show that people associate political themes such as environment, social security, and unemployment with left-wing parties, whereas themes like taxes, immigration, and crime are more often linked to right-wing parties. In a similar way, other research shows that the left-right divide can also be applied to the content of arguments about issues in political discourse. For example, left-wing parties favor policies that increase government spending and induce growth, while right-wing parties support policies inducing lower spending, balanced budgets, and lower inflation (see, e.g., [35], [36], [37]). Moreover, partisan cleavages can even be used to predict positions on new issues, such as on the EU integration [38]. In sum, these studies indicate that certain standpoints across a wide variety of political themes can be classified clearly on a left-right continuum. By using such different types of headings as issue frames in our VAA statements, we aim to study emphasis framing in such a way that generalizations beyond the level of individual items can be made.

The general finding in previous studies on emphasis framing is that people tend to adapt their opinion in the direction of a frame: If a frame motivates people to think positively about a course of action, they are inclined to agree with it; if a frame draws attention to negative aspects or consequences, people will more likely oppose (e.g., [6], [39], [40]). Therefore, we hypothesize that in a VAA context users follow the direction of the frame:

*H1*. *Right-wing headers lead to more right-wing opinions than conditions without a header above the question and left-wing headers lead to more left-wing opinions as compared to conditions without a header*.

### Political sophistication

An important issue is whether framing affects people’s reported attitudes across-the-board, or whether framing effects are systematically smaller or larger in size for particular groups of respondents. Dual-route theories of information processing such as the Elaboration Likelihood model (see [14], [15]) or Krosnick’s satisificing-optimizing model [13], have proposed that people who are motivated to think about an issue (e.g., people that are interested in an issue or for which the issue is personally relevant), as well as people with higher cognitive capacities (e.g., the higher educated) tend to process information about an issue more deeply. By doing so, they will probably be less susceptible to superficial characteristics of the way the information is conveyed, such as frame choices. And, although evidence for this claim is not always observed [41], several studies have indeed found framing effects to be related by variation in educational level [42], knowledge (see [39], [43], [44]) and the importance of the issue [45].

Comparable to these theories from the domains of social psychology and survey methodology, research in political decision making has indicated that one’s degree of political sophistication affects the way political information is organized and processed [46]. Political sophistication is related to both motivational factors, such as political interest, as well as to measures that are indicative of one’s cognitive capacities, such as educational level. Hence, political sophistication is a factor that combines the type of characteristics that are also deemed important indicators of attitude strength within social psychology and survey methodology (see [13], [47]). Therefore, we will test political sophistication as a moderator of possible effects of framing in the typical political attitude context of VAAs. In line with literature from various fields, we predict that:

H2. *Political sophistication moderates the framing effect such that the lower one’s political sophistication is*, *the stronger one is affected by framing variation*.

## Methods

### Design and materials

A randomized field experiment was carried out during the campaign for the 2014 Dutch municipality elections in Utrecht, a large Dutch city. For this experiment, we collaborated with the Voting Advice Application *Kieskompas*, which is the second most used VAA in the Netherlands (after *Stemwijzer*). The project description for this study was approved by the research director of the research institute UiL OTS of Utrecht University, by the registry and by the mayor of the city of Utrecht, by the director of Kieskompas.nl and a blanket approval of the Ethical Committee of Social Sciences at the Free University.

*Kieskompas* Utrecht was online for 30 days, during which 41,505 people accessed the website. People who visited the website were randomly assigned to one of five versions of the tool; they could stop filling out the VAA at any time they liked. We will elaborate on the five VAA versions below.

**Fig 1:**
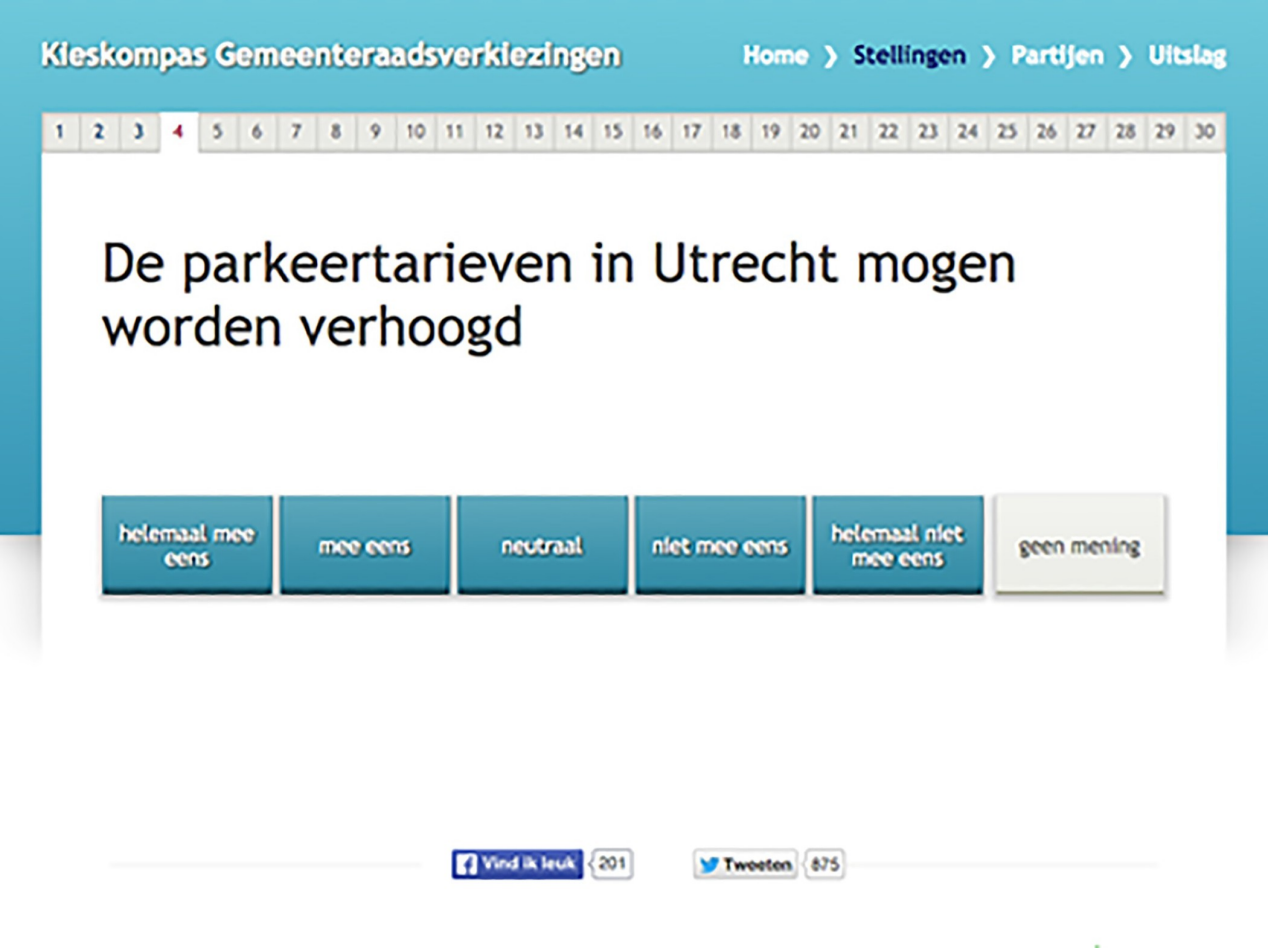
Example of a question from the benchmark version without headings. The question reads “The parking charges in Utrecht can be raised” with a response scale ranging from “completely agree” to “completely disagree” added with a “no opinion”-option.

The first VAA version was developed according to the standard procedure at *Kieskompas* (see for a discussion on this procedure: [8]). The VAA consists of 30 political attitude statements about various political issues and there were no headers presented above the questions (see Fig 1 for an example question).

Based on this first benchmark version, four alternative versions (version 2–5) of the VAA were created. These versions were manipulated according to a 2 x 2 experimental design of which one of the factors was the manipulation of issue framing that we report on here (with the levels: left-wing and right-wing), and the other manipulation concerned question polarity (with the levels: positive or negative wordings). As this second manipulation of question polarity did not interact with the manipulation of issue framing, we decided to report the effect of this factor elsewhere [19].

**Fig 2:**
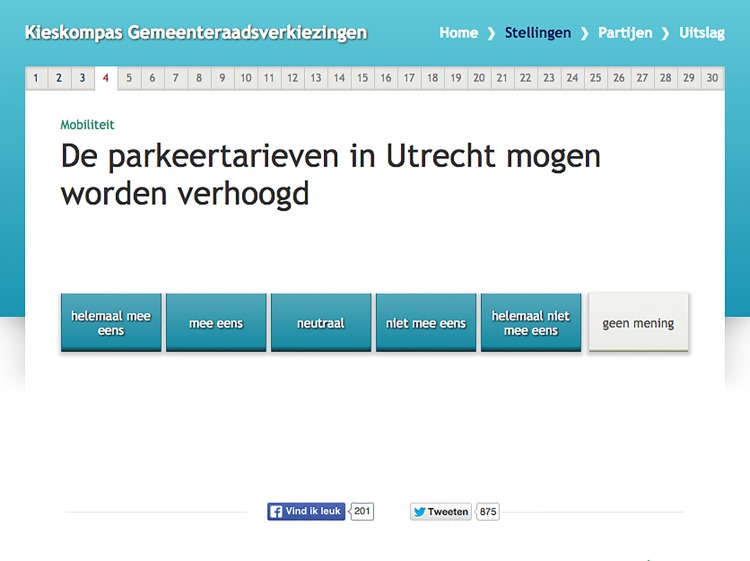
Example of a question with a left-wing heading “Natuur en milieu” (Nature and environment). The question reads “The parking charges in Utrecht can be raised” with a response scale ranging from “completely agree” to “completely disagree” added with a “no opinion”-option.

**Fig 3:**
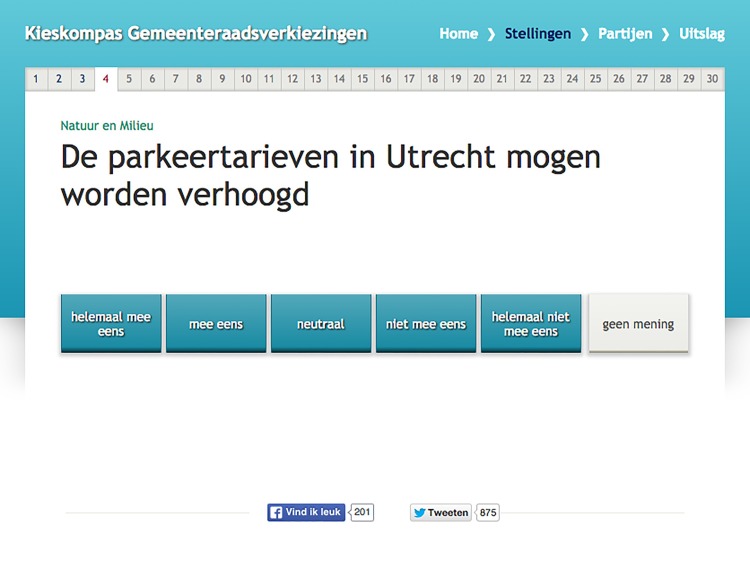
Example of a question with a right-wing heading “Mobiliteit” (Transportation). The question reads “The parking charges in Utrecht can be raised” with a response scale ranging from “completely agree” to “completely disagree” added with a “no opinion”-option.

Issue framing was manipulated by providing a header above the VAA statements. For each statement in versions 2 to 5, there was a header above the question and for a little more than half of these statements (*N* = 17), we were able to vary these headers between experimental conditions. To ensure ecological validity, we only used headers that VAA developer *Kieskompas* would normally use. We classified these existing headers the VAA builders generally used in their tools into two groups of left-wing and right-wing frames. We define left-wing headers to be headers that are associated with left-wing political parties (e.g., *nature and* environment, see Fig 2) and right-wing headers to be headers that are associated with right-wing political parties (e.g., *Transportation*, see Fig 3). As such we classified the headings *nature and environment*, *social policy* and *culture & education* to be left-wing frames, as these and comparable themes were clearly associated with left-wing parties in the study of associative ownership by [34]. Moreover, the themes *finances*, *economy*, *safety*, *transportation*, and *building & living*, were classified to be right-wing headers; the first three themes were associated with right-wing parties in the study by [34], the latter ones turned out to be suitable themes to distinguish between parties running in the Utrecht elections based on their party programs.

There were various reasons why we could not manipulate all statements in the VAA. One reason was that about half of the items did not scale onto the left-right axis, but on a progressive-conservative axis. Another reason was that sometimes we were unable to formulate relevant left- and right-wing headers for a statement.

In our experiment, the left- and right-wing frames were distributed across the different conditions in such a way that each VAA version approximately included an equal number of both left- and right-wing frames. This was done to assure that if the headers would influence people’s opinions in a consistent direction, the mean effect on the voting advice would be evened out. S1 Appendix provides an overview of the experimental materials.

### Procedure

When entering the *Kieskompas Utrecht* website, visitors were first asked to complete some demographic questions before answering the VAA questions (see S1 Appendix). Next, visitors indicated their opinions to 30 VAA statements on a five-point agree-disagree scale supplemented with a “no opinion” option. The voting advice, which followed after some optional questions about voting intentions, was based on the match between the VAA user’s answers and the stances of the political parties (see [8] for an elaboration).

### Measures

The dependent measure in our study was formed by the mean answers to the 17 manipulated attitude questions. We recoded these answers in such a way that a higher score indicates a more right-wing answer. Non-substantive answers were excluded from the analyses (a similar procedure was used in e.g., [19] and [41]).

To measure political sophistication, our moderator variable, we created an additive index combining a question about political interest with a question on educational level (compare: [19, 48]). This measurement of political sophistication was preferred to asking factual knowledge questions (as employed in e.g., [49]), because employing factual questions might have left respondents with the impression that they had to pass some sort of test in order to obtain a voting advice. In order to combine the questions on political interest (measured on a five-point scale) and educational level (measured on a seven-point scale) into one additive measure in which each measure receives an equal weight, we created two new variables with an equal number of scale points. We chose to recode education into 3 scale points, similar to the classification used by CBS (the Dutch Central Bureau of Statistics) and therefore recoded political interest into 3 scale points too. To create an index of political sophistication, we then combined these two variables into a new additive variable (compare: [19]).

### Participants

While 41,505 respondents accessed the *Kieskompas* website, the actual number of valid responses included in the analysis was far less. This is because only 28,741 users (69.25%) filled out the optional demographic questions on the first page of the tool that we used to measure political sophistication. Moreover, we cleaned our data, removing VAA users who are under 18 (and hence not eligible to vote), users who took less than two minutes to fill out the VAA, and users exposing straight-lining behavior providing the same answer to all political attitude questions in the tool. These cleaning criteria were also applied in a previous VAA study [19], and led to the exclusion of 1,337 respondents, leaving 27,404 respondents for the main analysis. We also re-ran the analyses described below in a data file that was not cleaned. Results for the full data file were comparable to what we report in the results section.

The mean age of respondents was 37.08 (*SD* = 13.96). Most VAA users were well-educated (78.4% had a Bachelor or Master degree), and most were fairly interested in politics (on a five-point scale: *M* = 3.3; *SD* = .84). The male-female ratio was about equal (Males: 49.3%; Females: 50.7%). A randomization check showed that VAA users in the various conditions did not differ significantly on age (*F*(4, 29353) = 0.79; *p* = .54), gender (χ^2^(4) = 3.62; *p* = .46), political sophistication (χ^2^(16) = 17.09; *p* = 38), educational level (χ^2^(24) = 14.59; *p* = .93) or interest in politics (*F*(4, 29015) = 0.66; *p* = .62). This means that there is no reason to assume that any possible framing effects are caused by *a priori* differences between groups.

### Manipulation check

As our manipulation of issue framing was quite subtle (see Figs 1–3), we checked whether the VAA users were likely to have noticed the headings above the statements. If so, we expected more processing time for the conditions with a header as compared to the benchmark version without headers. To test this, we compared the total time spent on the VAA between the benchmark version (without headers) and the experimental versions (including headers). The total time spent on the VAA is, of course, a rough measure of processing time, as it incorporates not only the time spent on the VAA statements, but also the time on the result screen and the additional demographic questions. Hence, if we were to find time differences between the benchmark version and the manipulated VAA versions (with the only difference that they include a header) for this noisy measure, this would be a strong indication that VAA users noticed the headers. Results show that VAA users spent about 16.5 seconds longer on the VAA when there were headers above the question (*M*_*no heading*_ = 451.69; *M*_*heading*_ = 468.25; *z* = 3.60; *p* < .001). VAA users thus spent approximately half a second longer per statement when headings are included, which is quite substantive when imagining that the answering of questions is a very fast process that takes about 3 seconds per question [50]. Therefore, there is no reason to assume that VAA-users might have missed the headers.

### Analyses

To test our hypotheses, we analyzed the VAA users’ answers in a multi-level model. In the model, the mean answer was estimated for questions without a header (i.e., questions related to the benchmark version). Subsequently, deviations from this mean answer were calculated for questions with a left-wing header, a right-wing header, for respondents with higher political sophistication, and the interactions between both left-wing and right-wing frames and political sophistication. To ensure a clean comparison between these different versions, we added question polarity as an additional term to the fixed part of the model. This final model fitted the data much better than a model that only included the issue framing effect (*z* = 111; *p* < .001).

The random part of the model accounts for the hierarchical structure of the data. As each respondent in our sample (*N* = 27,404) answered various manipulated questions (up to *N* = 17), each observation is nested within respondents and items at the same time. To account for this, the models distinguish between-item variance (e.g., because one item elicits more right-wing answers than another statement), between-respondent variance (e.g., because one respondent has more right-wing opinions than another respondent does), and a residue of error variance. Hence, cross-classified models are in operation (see also [51], [52]). For a formal representation of this model, we refer to S2 Appendix.

If there is an effect observed, we also classify the effect size by providing Cohen’s *d*. Cohen’s *d* is a measure for the effect size that relates the difference between conditions to the standard deviation [53]. In multi-level models such as the ones we have applied in the current research there are different variance components that can all be used separately or in combination with one another to calculate the effect size. In the current research we have used the between-item variance for calculating the effect size.

## Results

Table 1 shows the parameter estimates of the multi-level model used to analyze the data. Results indicate an effect of left-wing frames, (*z* = 6.82; *p* < .001; Cohen’s *d*_*items*_ = 0.88), an effect of right-wing frames (7.50; *p* < .001; Cohen’s *d*_*items*_ = 0.97), a main effect of political sophistication (*z* = 9.83; *p* < .001; Cohen’s *d*_*items*_ = 0.27), as well as interactions between both left- and right-wing frames and political sophistication (*z* = 7.29; *p* < .001; Cohen’s *d*_*items*_ = 0.24 and *z* = 7.71; *p* < .001; Cohen’s *d*_*items*_ = 0.25).

**Table 1:** The effect of issue framing and political sophistication on the answers across all statements.

	Mean (SE)	Change in Mean (SE)	S^2^_respondents_ (SE)	S^2^_items_ (SE)	S^2^_interaction_ (SE)
No Header	3.008 (0.058)		0.061 (0.001)	0.047 (0.016)	1.258 (0.003)
Right-wing frame		-0.191 (0.028)			
Left-wing frame		-0.210 (0.028)			
Sophistication		-0.059 (0.006)			
Right-wing frame * Sophistication		0.051 (0.007)			
Left-wing frame * Sophistication		0.054 (0.007)			

A higher score represents a more right-wing attitude. The model also included an additional term to filter out any possible side-effects of valence framing and allow for a clean comparison with the benchmark version without headers (0.570; SE = 0.004).

**Fig 4:**
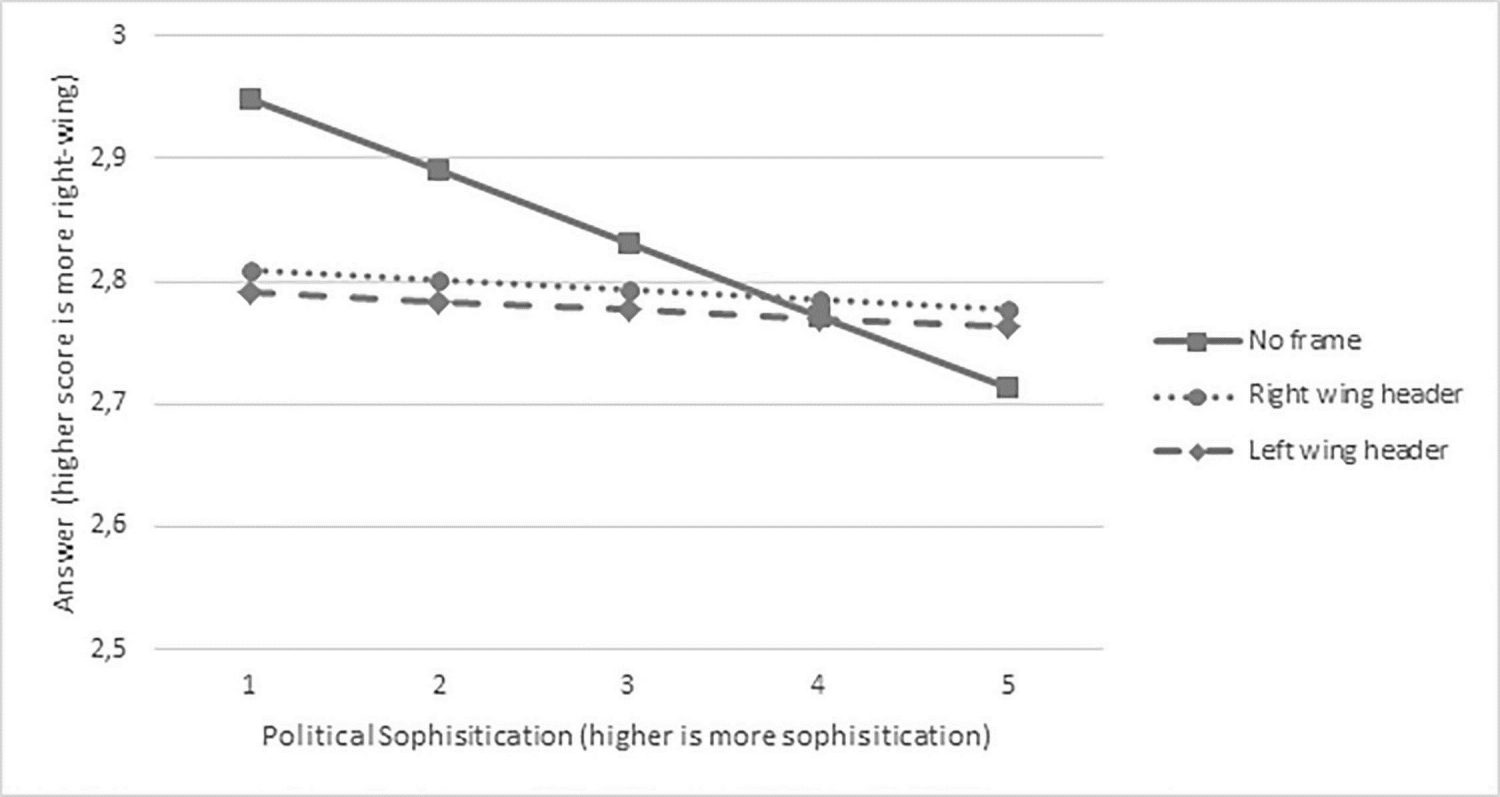
Visualization of the effect of framing on the answers for VAA users with lower and higher levels of political sophistication. The scale for political sophistication has been recoded to range from 1 (low) to 5 (high)).

To illustrate the pattern of the results, we visualized the parameter estimates in Fig 4. As can be seen there, providing any header at all (either left-wing or right-wing) triggers the lower sophisticated (i.e., those with lower levels of political sophistication) to provide more left-wing answers (Cohen’s *d*_*items+respondents*_ indicates a substantive effect size for the lowest two levels of sophistication; size ranges between 0.27 and 0.48), while for those with higher levels of political sophistication framing has no substantive effect on the answers (Cohen’s *d*_*items+respondents*_ < 0.20). Hence, even though the direction of the interaction is in line with our expectations (framing effects are larger for those with lower levels of political sophistication), the direction of the framing effect is not. We therefore ran several exploratory analyses to further understand these unexpected results.

### Explorations

First, we checked if our results may be due to a specific selection of left- and right-wing headings. As has been explained in the Method section, some of the left- and right-wing headers were derived from a previous study [34] and others from the party programs of the parties running in the 2014 Utrecht municipality elections. S3 Appendix includes an analysis of the results based on only those headings for which we had a motivation based on [34]. As can be read from the Appendix, results were comparable to what we have reported in Table 1.

Second, we analyzed the data for each statement separately to check whether there are individual statements that caused the overall effect to be in a different direction than expected. S4 Appendix displays the results of these models. As can be seen, 10 out of 17 statements show a pattern similar to the overall results: both left-wing and right-wing frames lead to more left-wing opinions for VAA users with lower levels of political sophistication. This implies that the overall pattern found is not bound to a specific pair of left- and right-wing frames, but really occurs for a wide range of headings (nature & environment vs. transportation; nature & environment vs. finance; culture and education vs. finance; nature and environment vs. building and living). In addition, there are two items (social policy vs. safety; social policy vs. finance) for which a reversed pattern is shown, in the sense that both the left-and right-wing frames lead to more right-wing opinions, but again, just for the those VAA users with lower levels of political sophistication. The remaining five questions do not show main effects of framing nor in interaction with political sophistication. Therefore, it seems unlikely that a specific pair of frames caused these results to occur and explaining the results probably lies in the interaction between the question content and the frame. We will return to this in the discussion section.

## Conclusion and discussion

This study examines issue framing effects in the context of Voting Advice Applications. In a large-scale (*N* = 27,404) randomized field experiment during local elections in the Netherlands, we varied the type of header above 17 VAA statements, systematically comparing a condition without a header to a condition with left-wing headers (e.g., *Nature and environment*) and a condition with right-wing headers (e.g., *Finance*).

Results indicate that issue framing affects the answers, but only for VAA users with lower levels of political sophistication. It is this group that supplies more left-wing answers when the statement includes any type of frame (so either a left-wing or a right-wing heading) relative to conditions where there is no header above the question at all. These findings are partially in line with our expectations: we did expect the framing effect to be larger for people with lower levels of political sophistication (based on e.g., [13], [14], [15]), but based on previous research (e.g., [6], [40]) the framing effect was expected to be in a different direction (right-wing header > no header > left-wing header).

These results raise the question as to why both left-wing and right-wing frames lead to more left-wing answers for the VAA users with lower levels of political sophistication. Our exploratory analyses showed that it was unlikely that one specific pair of frames caused the effect to be in a different direction than expected, as the same pattern was found for the majority of the individual items and for different sets of left- and right-wing frames. In addition, they also indicate that the overall findings are not due to interactions between the framing of the headers and the respondents’ self-perceived left-right status. Therefore, we think an explanation should be sought in the interaction between framing and the question content.

One such explanation is that in some contexts the right-wing frames were viable to multiple interpretations, including (unintended) left-wing ones. For example, consider the right-wing header *Finance* above the statement *The municipality should invest extra money to fight children’s language deficiencies*. In terms of the ideological left-right divide, this frame was supposed to trigger the right-wing argument that fighting language deficiencies costs (too much) community tax and that citizens are responsible for their own language acquisition. However, this same heading of *Finance* for this specific question may have also triggered that the government invests in the weaker groups in society. Put differently, the minimal left- and right-wing frames may not have always triggered the exact left- and right-wing arguments we expected them to evoke; in particular the headings *Finance* and *Economy*, that comprised the right-wing frames for 11 out of the 17 statements, may have triggered arguments related to government finance as well as those related to the financial situation of individual citizens in society. To further investigate this explanation, we propose a future study in which not only the time spent on the VAA versions with and without headings is compared, but the frames are also pretested to find out the exact arguments they elicit. A qualitative research method such as thought listing tasks [54] would be suitable for such a study.

The current research adds to existing research on framing in various ways. First, it shows evidence for effects of emphasis framing when it is manipulated in a subtle way without potential confounds playing a role.

Second, it finds evidence for the existence of these framing effects in the new context of VAAs. Users of VAAs have other objectives for filling out survey questions (or statements) than respondents in a regular survey: they take the test spontaneously and out of interest rather than feeling obliged. Even in such a presumably high-elaboration context and even when applying a very subtle manipulation, the framing of statements matters.

Third, political sophistication has been shown to be an important moderator of framing effects in a political attitude context. Using an operationalization that combined political interest with the respondent’s educational level, we were able to demonstrate that framing in headings above VAA statements only affects the lower sophisticated. This is probably due to the fact that the lower sophisticated hold weak attitudes about the political issues at stake (see [13], [14], [15]). Therefore, they will have to come up with an opinion on the spot for most of the 30 VAA statements. Due to the lack of (strong) existing attitudes, they will rely on superficial cues of the question context, such as the heading above the question, to formulate arguments and report an opinion. This causes the framing of the heading to affect the attitudes reported by this group.

A variation of this explanation can be formulated when we also incorporate literature from media framing effects. One of the possible explanations for issue framing effects that has been suggested is that framing can change the content of the beliefs about an issue, adding new considerations and links between considerations. This may, in turn, change the balance of considerations in people’s minds, altering the attitudes people hold [55]. Hence, such an explanation also presumes a dual process model, but here the framing effects that occur for the lower sophisticated do not seem to be born out of superficial processing.

Future research should establish which cognitive mechanism causes framing effects to arise in the specific political attitude context of VAAs. One way to do this is to perform a new experimental framing study but to add extra conditions in which participants are distracted while filling out the VAA (for example by playing loud music in the background). If shallow processing causes framing effects to arise, we expect larger framing effects for the lower sophisticated under distracting conditions than under non-distracting conditions. Moreover, we may expect the higher sophisticated to expose framing effects under these conditions too.

Besides theoretical implications the current research also has practical consequences, although these consequences depend on how problematic one perceives the framing effects observed to be. It can be argued that if people’s opinions are only partly dependent on the way issues are framed, this raises concerns regarding the competence of citizens to fulfill the role they have been assigned in a democracy. Two broadly shared criteria of citizen competence are that people’s political opinions should not be subject to “arbitrary aspects of how an issue or problem is described” or to “elite manipulation” ([21]: pp. 232–233). In line with this view, the results of the current study should lead to the practical advice to frame as little as possible, and hence, not to use headings in VAA statements.

One can also argue that the effect of framing on democracy can only be assessed if the thriving cognitive mechanisms behind these effects are unraveled. Framing effects may be viewed as a blessing rather than a curse if they are the result of the lower sophisticated spending more time and cognitive energy thinking about the issues at stake. If this were indeed the case, our practical advice would be to include left-wing frames as well as right-wing frames in a VAA, to randomly present these left- and right-wing frames to users and to do this in such a way that each user always receives an equal number of right- and left-wing frames. After all, the aim of VAA developers is to inspire users to think about the issues at stake.

## Supporting information

S1 Appendix Experimental materials in Dutch and in a rough English translation. (DOCX)

S2 Appendix Formalization of multi-level models. (DOCX)

S3 Appendix Parameter estimates of the model including statements for which the headings were based on Walgrave et al. (2012). (DOCX)

S4 Appendix Parameter estimates of the models per question. (DOCX)
