# Correction: Issue framing in online voting advice applications: The effect of left-wing and right-wing headers on reported attitudes

**DOI:** 10.1371/journal.pone.0216969

**Published:** 2019-05-09

**Authors:** The PLOS ONE Staff

The Data Availability statement for this paper is incorrect. The publisher apologizes for the error. The correct statement is: Data underlying the study is available in the Dataverse repository (https://dataverse.nl/dataset.xhtml?persistentId=hdl:10411/MJW4OG).
