# Supplementary material for: Issue framing in online voting advice applications: The effect of left-wing and right-wing headers on reported attitudes
Source: PLoS One. 2019 Feb 21;14(2):e0212555. doi: 10.1371/journal.pone.0212555 (PMC6383922; doi:10.1371/journal.pone.0212555)
Supplement: S1 Appendix — (DOCX) [file pone.0212555.s001.docx]

S1 Appendix. Experimental materials in Dutch and in a rough English translation.

Prior to answering the VAA statement, users could optionally answer the following 4 questions:

1. Wat is uw geslacht? Man/ Vrouw
   What is your gender? Male/Female
2. Wat is uw geboortejaar? (lijst van jaren)
   What is your year of birth? (list of years)
3. Wat is uw hoogst genoten opleiding? (lijst van opleidingsniveau’s)
   What is the level of your highest completed education? (list of educational levels)
4. Hoeveel interesse heeft u in de politiek? (5-puntsschaal van heel veel – heel weinig interesse)
   How much interest do you have in politics? (5 point scale ranging from a lot of interest to very little interest).

Next they answered 30 attitude questions in one of the experimental versions; these have been displayed in Table A1 and A2.

*Table A1:* Statements and accompanying left- and right-wing frames in Dutch (versions in which this frame was used between brackets)

| **№** | **Statement** | **Left-wing frame** | **Right-wing frame** |
| --- | --- | --- | --- |
| 1 | Er moet een verbreding komen van de A27 bij Amelisweerd | Natuur & Milieu  (1 & 3) | Mobiliteit (2 & 4) |
| 2 | De parkeertarieven in Utrecht mogen worden verhoogd | Natuur & Milieu (1& 3) | Mobiliteit (2 & 4) |
| 3 | De meest vervuilende auto’s (ouder dan Diesel Euro 3 en Benzine Euro 0) moeten uit de binnenstad worden geweerd | Natuur & Milieu (2 & 4) | Mobiliteit (1 & 3) |
| 4 | Het budget om de leefbaarheid in wijken te verbeteren (het leefbaarheidsbudget) moet worden afgeschaft | Sociaal beleid (2 & 4) | Financiën (1 & 3) |
| 5 | In achterstandswijken mogen sociale huurwoningen worden gesloopt | Sociaal beleid (1 & 3) | Veiligheid (2 & 4) |
| 6 | Op welzijnswerk mag worden bezuinigd | Sociaal beleid (1 & 3) | Financiën (2 & 4) |
| 7 | Er mogen woningen worden gebouwd in de polder Rijnenburg | Natuur & Milieu  (1 & 3) | Bouwen & Wonen  (2 & 4) |
| 8 | De gemeente moet geld investeren in een fietsbrug tussen Oog in Al en Leidsche Rijn over het Amsterdam-Rijnkanaal | Natuur & Milieu  (2 & 4) | Financiën (1 & 3) |
| 9 | De gemeente moet extra geld investeren om taalachterstanden bij kinderen tegen te gaan | Cultuur & Onderwijs (1 & 3) | Financiën ( 2 & 4) |
| 10 | De afvalstoffenheffing moet worden afgeschaft | Natuur & Milieu (2 & 4) | Financiën (1 & 3) |
| 11 | Op kunst en cultuur mag worden bezuinigd | Cultuur & Onderwijs  (2 & 4) | Financiën (1 & 3) |
| 12 | Voor milieumaatregelen mag een belastingverhoging plaatsvinden | Natuur & Milieu (1 & 3) | Financiën (2 & 4) |
| 13 | De gemeente mag bedrijven dwingen tot het nemen van energiebesparende maatregelen | Natuur & Milieu  (2 & 4) | Economie (1 & 3) |
| 14 | Op industrieterrein Lage Weide mag een windmolenpark komen | Natuur & Milieu  (1 & 3) | Bouwen & Wonen  (2 & 4) |
| 15 | De gemeente moet meer geld uittrekken voor armoedebestrijding, ook als hiervoor de belastingen moeten worden verhoogd | Sociaal beleid  (1 & 4) | Economie (2 & 3) |
| 16 | De gemeente kan beter tijdelijk schulden maken dan bezuinigen op sociale voorzieningen | Sociaal beleid (1 & 3) | Economie (2 & 4) |
| 17 | Iedere bijstandsontvanger moet verplicht vrijwilligerswerk doen, of worden gekort op zijn uitkering | Sociaal beleid (1 & 4) | Economie  (2 & 3) |

*Table A2:* Statements and accompanying left- and right-wing frames in a rough English translation (versions in which this frame was used between brackets)

| **№** | **Statement** | **Left-wing frame** | **Right-wing frame** |
| --- | --- | --- | --- |
| 1 | The A27 highway should be broadened at Amelisweerd | Nature & Environment  (1 & 3) | Transportation (2 & 4) |
| 2 | Parking charges should be raised in the city of Utrecht | Nature & Environment (1& 3) | Transportation (2 & 4) |
| 3 | The cars polluting most (older than EURO 3 for diesel and EURO 0 for gasoline) should be banned from the city center | Nature & Environment (2 & 4) | Transportation (1 & 3) |
| 4 | The budget for improving neighbourhood liveability (the liveability budget) should be abolished | Social policy (2 & 4) | Finances (1 & 3) |
| 5 | In disadvantaged neighborhoods, the municipality may tear down social housing | Social policy (1 & 3) | Safety (2 & 4) |
| 6 | The municipality can cut down on social work | Social policy (1 & 3) | Finances (2 & 4) |
| 7 | The municipality can build houses in the Rijnenburg polder | Nature & Environment  (1 & 3) | Building & Living  (2 & 4) |
| 8 | The municipality should invest money to build a bicycle bridge over the Amsterdam-Rijn canal between Oog in Al and Leidsche Rijn | Nature & Environment  (2 & 4) | Finances (1 & 3) |
| 9 | The municipality should invest extra money to fight children’s language deficiencies | Culture & Education (1 & 3) | Finances  ( 2 & 4) |
| 10 | Waste charges should be abolished | Nature & Environment (2 & 4) | Finances (1 & 3) |
| 11 | The municipality may cut down on art and culture | Culture & Education (2 & 4) | Finances (1 & 3) |
| 12 | Taxes may be increased for measures for the environment | Nature & Environment (1 & 3) | Finances (2 & 4) |
| 13 | The municipality may force businesses to take energy-saving measures | Nature & Environment  (2 & 4) | Economy (1 & 3) |
| 14 | A wind park can be built at the Lage Weide industrial zone | Nature & Environment (1 & 3) | Building & Living  (2 & 4) |
| 15 | The municipality should spend more money to fight poverty, even if taxes should be increased for this | Social policy  (1 & 4) | Economy (2 & 3) |
| 16 | It's better if the municipality has temporary deficits instead of having to cut down on social services | Social policy (1 & 3) | Economy (2 & 4) |
| 17 | Everyone on social benefits should be forced to do volunteering work, or their benefits will be cut back | Social policy  (1 & 4) | Economy  (2 & 3) |

After optionally judging political parties and receiving a voting advice, VAA users saw a pop-up survey with some more questions they could optionally answer.
