# Supplementary material for: Issue framing in online voting advice applications: The effect of left-wing and right-wing headers on reported attitudes
Source: PLoS One. 2019 Feb 21;14(2):e0212555. doi: 10.1371/journal.pone.0212555 (PMC6383922; doi:10.1371/journal.pone.0212555)
Supplement: S2 Appendix — (DOCX) [file pone.0212555.s002.docx]

S2 Appendix: Formalization of multi-level models

In Equation 1, the model used for analyzing the main effect of issue framing is formalized. In this model, *Y_(jk)_* the score of individual *j* (*j* = 1, 2…27,404) on question *k* (*k* = 1, 2,…17) on a five-point scale that is recoded such that a higher score represents a more right-wing attitude. In the fixed part of the model a constant (CONS) estimates the average score (β_1_) for respondents in the benchmark condition. This score is allowed to vary between respondents (one respondent may provide more no-opinion answers than another respondent), items (one item may generate more no-opinion answers than another item), and due to the interaction between respondent and item (terms: *u*_1j_, *v*_0_*_k_* , and *w*_j_*_k_*). The item and respondent variance are estimated simultaneously, which means that a cross-classified model is in operation. All residuals are normally distributed with an expected value of zero, and a variance of respectively S^2^*_u_*_1_*_j_*_,_ S^2^*_v_*_0_*_k_* and S^2^*_w_*_j_*_k_*.
 To estimate the effect of right and left-wing frames, dummy variables (D_RIGHT-WING etc.) are created that can be turned on if the observation matches the prescribed type. Using these dummies, two deviations (Dβ_2_ and Dβ3) are estimated, that indicate how the mean score is changed when a right or left-wing frame is added. In the same way, we added terms to the fixed part of the model to estimate the effect of political sophistication, as well as the interaction between both right- and left-wing frames and political sophistication. Finally, the model includes a term estimating the change in scores of the question polarity is different from the polarity of the question in the benchmark version (Dβ_7_). The adding for this control variable is necessary to allow a clean comparison between the benchmark version that is not manipulation for question polarity.

Equation 1:

*Y_(jk)_* = CONS (β_1_ + *u*_1j_ + *v*_0_*_k_* + *w*_j_*_k_) +* D_RIGHT-WING (Dβ_2_) + D_LEFT-WING (Dβ_3_) + D_SOPHISTICATION (Dβ_4_) + D_RIGHT-WING *SOPHISTICATION (Dβ_5_) + D_LEFT-WING * SOPHSITICATION (Dβ_6_) + D_DIFFERENTVALENCE (Dβ_7_)
