# Supplementary material for: Issue framing in online voting advice applications: The effect of left-wing and right-wing headers on reported attitudes
Source: PLoS One. 2019 Feb 21;14(2):e0212555. doi: 10.1371/journal.pone.0212555 (PMC6383922; doi:10.1371/journal.pone.0212555)
Supplement: S3 Appendix — (DOCX) [file pone.0212555.s003.docx]

**S3 Appendix: Parameter estimates of the model including statements for which the headings were based on Walgrave et al. (2012).**

Table A3. The effect of issue framing and political sophistication on the answers across statement 4, 5, 6, 8, 9, 10, 11, 12 and 13

|  | Mean (SE) | Change in Mean (SE) | S^2^_respondents_ (SE) | S^2^_items_ (SE) | S^2^_interaction_ (SE) |
| --- | --- | --- | --- | --- | --- |
| No Header | 2.876 (0.082) |  | 0.064  (0.001) | 0.072 (0.030) | 1.132 (0.003) |
| Right frame |  | -0.095 (0.030) |  |  |  |
| Left frame |  | -0.111 (0.030) |  |  |  |
| Sophistication |  | -0.038 (0.006) |  |  |  |
| Right frame * Sophistication |  | 0.030 (0.007) |  |  |  |
| Left frame * Sophistication |  | 0.032 (0.007) |  |  |  |

A higher score represents a more right-wing attitude. The model also included an additional term to filter out any possible side-effects of valence framing and allow for a clean comparison with the benchmark version without headers (0.720; SE =0.005).
