# Supplementary material for: Issue framing in online voting advice applications: The effect of left-wing and right-wing headers on reported attitudes
Source: PLoS One. 2019 Feb 21;14(2):e0212555. doi: 10.1371/journal.pone.0212555 (PMC6383922; doi:10.1371/journal.pone.0212555)
Supplement: S4 Appendix — (DOCX) [file pone.0212555.s004.docx]

**S4 Appendix: Parameter estimates of the models per question.**

| Statement (*N*) | Constant (SE) | Right-wing frame (SE) | Left-wing frame (SE) | Valence framing (SE) | Sophistica-tion (SE) | Right frame * Sophistica-tion (SE) | Left frame * Sophistica-tion (SE) | S^2^ (SE) |
| --- | --- | --- | --- | --- | --- | --- | --- | --- |
| 1  (N = 25,495) | 2.879  (0.105) | -0.469 (0.128) | -0.369 (0.128) | 0.918 (0.018) | -0.085 (0.020) | 0.094  (0.025) | 0.080  (0.025) | 1.612  (0.014) |
| 2  (N = 26,921) | 4.925  (0.091) | -1.119 (0.111) | -1.097 (0.111) | -1.559 (0.016) | -0.220 (0.018) | 0.212  (0.022) | 0.209  (0.022) | 1.302  (0.011) |
| 3  (N = 26,787) | 3.459  (0.097) | -0.639 (0.120) | -0.667 (0.119) | 0.778  (0.017) | -0.138 (0.019) | 0.118  (0.023) | 0.124  (0.023) | 1.492  (0.013) |
| 4  (N = 26,897) | 2.188  (0.085) | -0.027 (0.105) | 0.041 (0.104) | 1.463 (0.015) | 0.008  (0.017) | 0  (0.021) | -0.012 (0.020) | 1.159 (0.010) |
| 5  (N = 26,832) | 2.160  (0.103) | 0.892 (0.127) | 0.787 (0.127) | 0.019 (0.018) | 0.144  (0.020) | -0.168 (0.025) | -0.152 (0.025) | 1.703  (0.015) |
| 6  (N = 27,042) | 1.849  (0.088) | 0.580  (0.108) | 0.549  (0.108) | 1.084 (0.015) | 0.092  (0.017) | -0.101 (0.021) | -0.087 (0.021) | 1.235  (0.011) |
| 7  (N = 24,824) | 2.201  (0.126) | 0.186 (0.157) | -0.103 (0.158) | 0.902  (0.023) | -0.010 (0.025) | -0.023 (0.031) | 0.027  (0.031) | 2.546 (0.023) |
| 8  (N = 25,089) | 2.972  (0.090) | -0.484 (0.110) | -0.468  (0.110) | 0.806  (0.015) | -0.077 (0.018) | 0.102  (0.022) | 0.091  (0.022) | 1.186 (0.011) |
| 9  (N = 27,001) | 2.736 (0.079) | -0.528 (0.097) | -0.387 (0.098) | 1.461 (0.014) | -0.97  (0.016) | 0.100  (0.019) | 0.087  (0.019) | 0.996  (0.009) |
| 10  (N = 26,589) | 3.281  (0.096) | -0.772 (0.118) | -0.803 (0.118) | 1.008  (0.017) | -0.172 (0.019) | 0.147  (0.023) | 0.150  (0.023) | 1.469  (0.013) |
| 11  (N = 27,278) | 3.079  (0.097) | -0.277 (0.119) | -0.373 (0.118) | 0.437 (0.017) | -0.081 (0.019) | 0.066  (0.023) | 0.085  (0.023) | 1.492  (0.013) |
| 12  (N = 26,581) | 3.710  (0.086) | -1.051 (0.105) | -0.988 (0.106) | 0.084  (0.015) | -0.164 (0.017) | 0.202  (0.021) | 0.194  (0.021) | 1.144  (0.010) |
| 13  (N = 27,050) | 2.499  (0.084) | -0.390 (0.104) | -0.341 (0.103) | 0.965  (0.014) | -0.028 (0.017) | 0.074  (0.020) | 0.064  (0.020) | 1.115  (0.010) |
| 14  (N = 25,987) | 2.792  (0.091) | -0.428 (0.112) | -0.473 (0.112) | 1.205 (0.022) | -0.085 (0.018) | 0.088  (0.022) | 0.100  (0.022) | 1.263  (0.011) |
| 15  (N = 26,714) | 3.219  (0.085) | 0.049 (0.104) | 0.011  (0.105) |  | -0.054 (0.017) | -0.004 (0.020) | 0  (0.020) | 1.136  (0.010) |
| 16  (N = 26,534) | 2.505  (0.086) | -0.012 (0.106) | -0.076 (0.105) |  | 0.098  (0.017) | 0.004  (0.021) | 0.019  (0.021) | 1.149  (0.010) |
| 17  (N = 27,388) | 3.765  (0.101) | 0.173  (0.123) | 0.192 (0.123) |  | -0.070 (0.020) | -0.030 (0.024) | -0.043 (0.024) | 1.621  (0.014) |

*Note* Question 15-17 do not include a parameter estimate for valence as these questions did not contain a second manipulation of issue framing
